# Supplementary material for: Non-equilibrium dissipative supramolecular materials with a tunable lifetime
Source: Nat Commun. 2017 Jul 18;8:15895. doi: 10.1038/ncomms15895 (PMC5520059; doi:10.1038/ncomms15895)
Supplement: Supplementary Information [file ncomms15895-s1.pdf]

Type of file: PDF
Size of file: 0 KB
Title of file for HTML: Supplementary Information
Description: Supplementary Figures, Supplementary Tables, Supplementary Notes and Supplementary Methods

Type of file: PDF
Size of file: 0 KB
Title of file for HTML: Peer Review File
Description:

Supplementary Note 1. In chemical reaction networks that use E (glutamic acid) as a precursor, we found significant N-acyl urea side product. Although that did not affect material properties, it did accumulate as the cycle progressed. The amount of N-Acyl urea we found was never greater than 2% of the amount of EDC added. For instance, after the addition of 10 mM fuel to 10 mM solution of precursor the N-acyl urea detected for Fmoc-E was 2%. The percentage of N-acyl urea was less than 0.5% for Fmoc-AAE and less than 0.1% for Fmoc-AVE.

Supplementary Note 2. A kinetic model was written using Matlab in which the reactions below were described. The concentrations of each reactant were calculated for every 1 second in the cycle.

Reaction 0 (k_0)

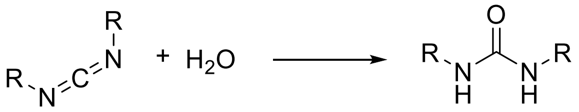

Direct hydrolysis of carbodiimide with a first order rate constant of $1.3x10^{-5}$ sec^{-1} as determined by HPLC (See Supplementary Figure 1c) and implies that in most experiments, this reaction is irrelevant.

Reaction 1 (k_1)

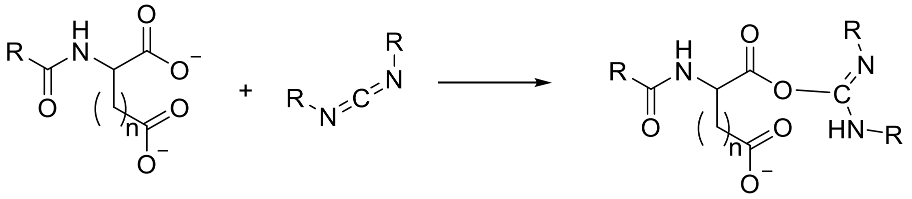

Formation of O-acylisourea by reaction with EDC. This second order rate constant was dependent on the nature of the precursor. The rate constant was determined for each precursor by HPLC, by monitoring the EDC consumption.

Reaction (k_2)

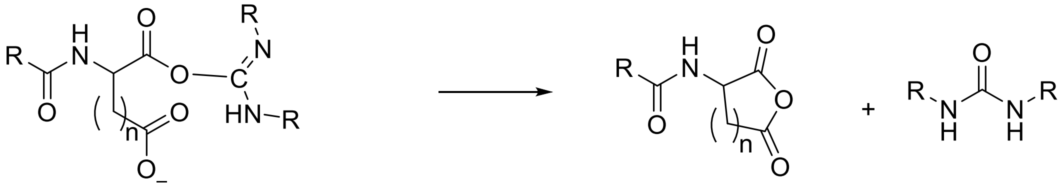

Formation of anhydride with a first order rate constant. This rate constant could not be determined because the O-acylisourea was never observed. It was therefore set to be twice the rate of k_1. As a result, the O-acylisourea did never reach concentrations over 1 μM in the model.

Reaction 3 (k_3)

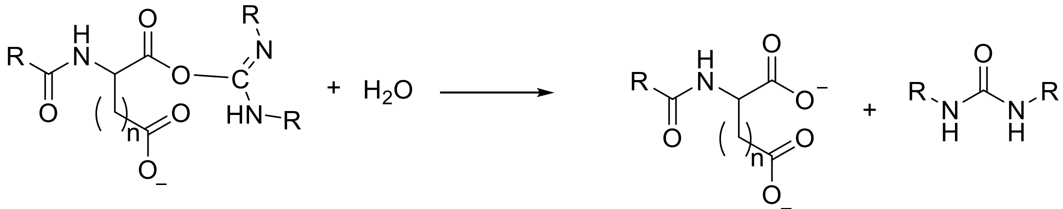

Direct hydrolysis of O-acylisourea (unwanted side reaction). This reaction rate could not be obtained because the O-acylisourea was not observed. The ratio of k_2 and k_3 (anhydride formation and competing direct hydrolysis of O-acylisourea) was varied to fit the HPLC data for several concentration of $[fuel]_0$ and $[di\text{-}acid]_0$.

Reaction 4 (k_4 and k_5)

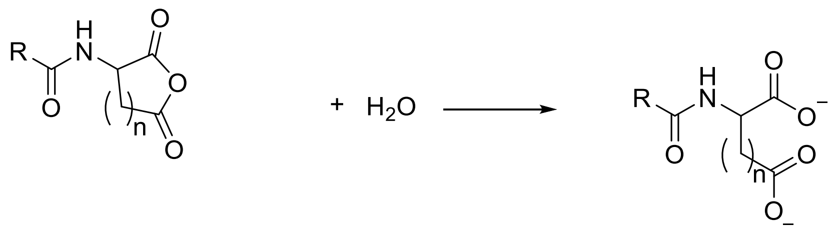

Hydrolysis of anhydride proceeded with a (pseudo)-first order rate as determined by HPLC. We found that for Fmoc-D, this rate was slowed down two orders of magnitude if assemblies were formed. When small amounts of fuel were added and no assemblies were formed, the HPLC data could be fitted with k_4. However, when a greater batch of fuel was added (3mM or more), we could not fit the data and had to implement a 1st order k_5 for the hydrolysis reaction in the models that acts when the concentration anhydride was >0.08 mM. In other words, the assemblies exerted negative feedback on their own degradation (they slowed down hydrolysis).

For Fmoc-E, similar behavior was found. At low fuel concentrations, the model could fit the data well with k_4. As soon as we added 5 mM or more fuel, the concentration anhydride reached values higher than 0.13 mM and the data could not be fit. Here, the behavior was somewhat different than for Fmoc-D. We needed to implement a 0th order k_5 that only acted when the concentration anhydride was > 0.13 mM in order to fit the data well. This can be rationalized by a mechanism where the assemblies exclude water from the anhydrides, protecting them from degradation. They do that such that hydrolysis is limited by the solubility of the anhydride.

Supplementary Table 1: Table of k values used in kinetic model

	k_1 (M^{-1} x sec^{-1})	k_2	k_3	k_4 (sec^{-1})	Half-life of anhydride (calculated by: $\ln(2)/k_4$)	k_5 (M x sec^{-1})	Half-life of anhydride (calculated by: $\ln(2)/k_5$)
Order	2nd	1st	1st	1st		0th or 1st	
Fmoc-D	$1.2x10^{-1}$	$2*k_1$	$4.0*k_1$	$1.4x10^{-2}$	$t_{1/2}$ =0.8 min	$3.8x10^{-4}$ (1st order)	$t_{1/2}$ =30 min
Fmoc-AVD	$2.0x10^{-1}$	$2*k_1$	$2.5*k_1$	$3.8x10^{-2}$	$t_{1/2}$ =0.3 min	NA	NA
Fmoc-AAD	$0.95x10^{-1}$	$2*k_1$	$2.5*k_1$	$2.8x10^{-2}$	$t_{1/2}$ =0.4 min	NA	NA
Fmoc-E	$0.50x10^{-1}$	$2*k_1$	$2.0*k_1$	$0.75x10^{-2}$	$t_{1/2}$ =1.5 min	$1.5x10^{-6}$ (0th order)	NA Depends on [anhydride]
Fmoc-AVE	$1.5x10^{-1}$	$2*k_1$	$2.5*k_1$	$1.1x10^{-2}$	$t_{1/2}$ =1.1 min	NA	NA
Fmoc-AAE	$0.40x10^{-1}$	$2*k_1$	$2.9*k_1$	$1.3x10^{-2}$	$t_{1/2}$ =0.9 min	NA	NA

Supplementary Table 2: Characterization of precursors

name	purity [%]	structure	mass calculated [g/mol]	mass found [g/mol]	retention time [min]
Fmoc-D	Commercial: 95%	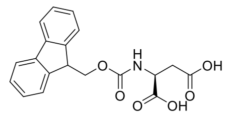	Mw = 355.11 $C_{19}H_{17}NO_6$	378.1 $[Mw+Na]^+$	6.13
Fmoc-E	Commercial: 95%	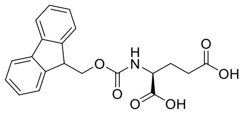	Mw = 369.12 $C_{20}H_{19}NO_6$	392.1 $[Mw+Na]^+$	6.14
Fmoc-AAE	97	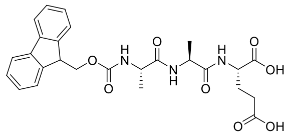	Mw = 511.20 $C_{26}H_{29}N_3O_8$	534.2 $[Mw+Na]^+$	5.64
Fmoc-AAD	98	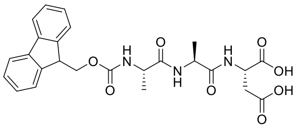	Mw = 497.18 $C_{25}H_{27}N_3O_8$	520.3 $[Mw+Na]^+$	5.62
Fmoc-AVE	98	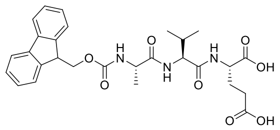	Mw = 539.23 $C_{28}H_{33}N_3O_8$	562.2 $[Mw+Na]^+$	6.21

name	purity [%]	structure	mass calculated [g/mol]	mass found [g/mol]	retention time [min]
Fmoc-AVD	99	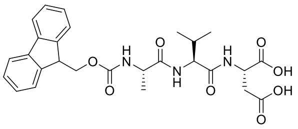	Mw = 525.21<br>$C_{27}H_{31}N_3O_8$	548.2<br>$[Mw+Na]^+$	6.21

Supplementary Table 3: Characterization of main products of the chemical reaction network

name	structure	mass calculated [g/mol]	mass found [g/mol]	retention time [min]
Fmoc-D-anhydride	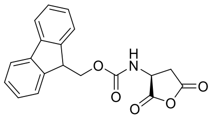	Mw = 337.10<br>$C_{19}H_{15}NO_5$	360.0<br>$[Mw+Na]^+$	7.88
Fmoc-E-anhydride	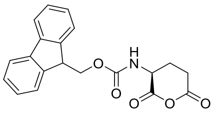	Mw = 351.11<br>$C_{20}H_{17}NO_5$	373.9<br>$[Mw+Na]^+$	7.68
Fmoc-AAE-anhydride	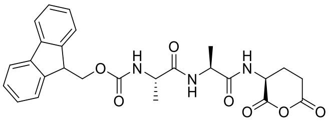	Mw = 493.18<br>$C_{26}H_{27}N_3O_7$	516.2<br>$[Mw+Na]^+$	6.68
Fmoc-AAD-anhydride	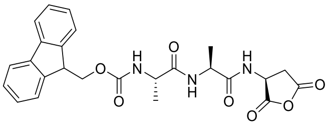	Mw = 479.17<br>$C_{25}H_{25}N_3O_7$	502.1<br>$[Mw+Na]^+$	6.93
Fmoc-AVE-anhydride	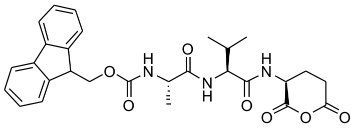	Mw = 521.22<br>$C_{25}H_{25}N_3O_7$	544.3<br>$[Mw+Na]^+$	7.32
Fmoc-AVD-anhydride	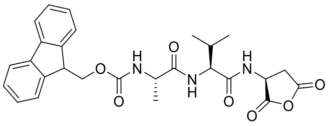	Mw = 507.20<br>$C_{27}H_{29}N_3O_7$	530.2<br>$[Mw+Na]^+$	7.54

Supplementary Table 4: Characterization of the side products of the chemical reaction network

Name	side product	mass calculated [g/mol]	mass found [g/mol]	retention time [min]
Fmoc-E-N-acyl-urea	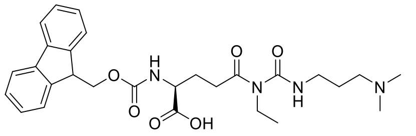	Mw = 524.26 $C_{28}H_{36}N_4O_6$	525.5 [Mw+H]	6.97
Fmoc-AAE-N-acyl-urea-1	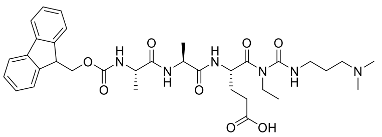	Mw = 666.33 $C_{34}H_{46}N_6O_8$	667.4 [Mw+H]	6.52
Fmoc-AAE-N-acyl-urea-2	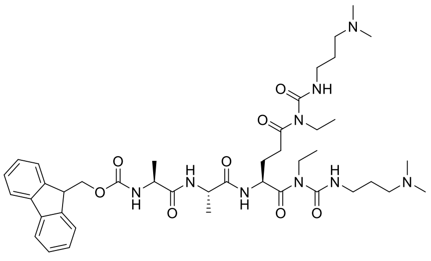	Mw = 821.48 $C_{42}H_{63}N_9O_8$	822.2 [Mw+H]	6.60
Fmoc-AVE-N-acyl-urea-1	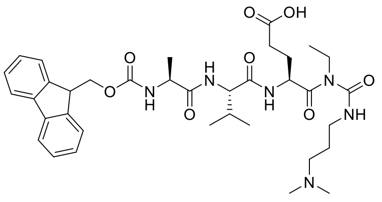	Mw = 694.37 $C_{36}H_{50}N_6O_8$	695.5 $[Mw+H]^+$	6.84
Fmoc-AVE-N-acyl-urea-2	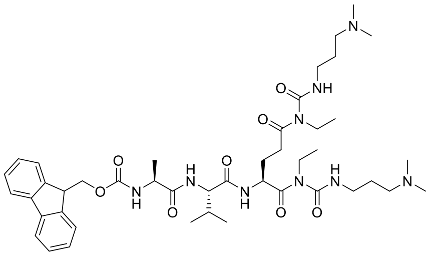	Mw = 849.51 $C_{44}H_{67}N_9O_8$	850.6 $[Mw+H]^+$	7.12

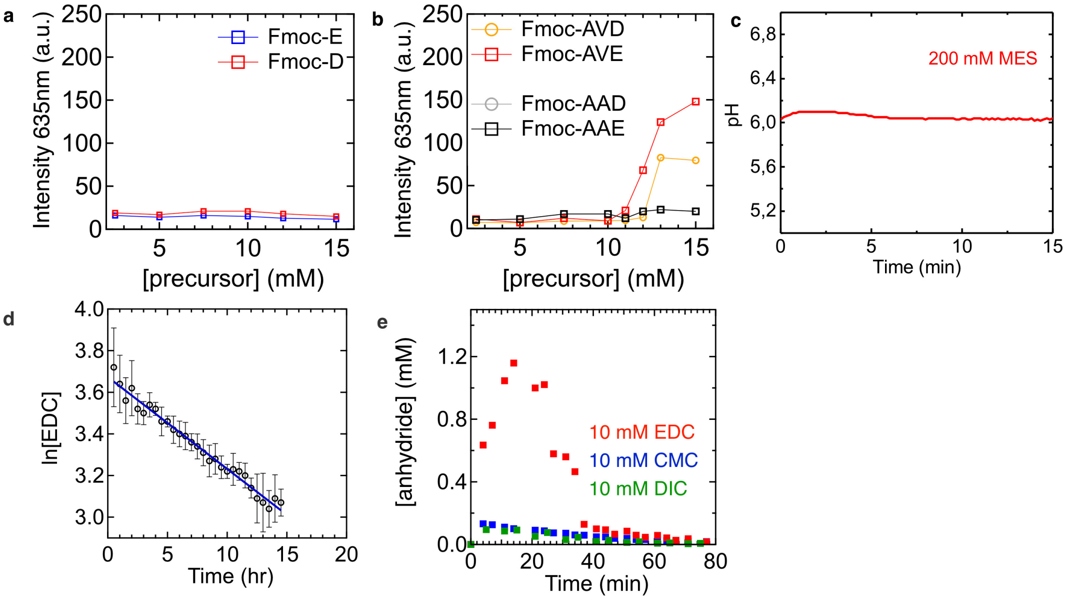

Supplementary Figure 1. Buffer stability, background hydrolysis of fuel and responses of Fmoc-E to different fuels. a, b, *Nile Red fluorescence intensity at 635 nm against concentration precursor. The intensity of the solvatochromic dye serves as an indication for self-assembly and was not observed for concentrations below 15 mM Fmoc-D or Fmoc-E* ***(a)****, or below a concentration of 12 mM for Fmoc-AVD, Fmoc-AVE, Fmoc-AAD or Fmoc-AAE* ***(b)****.* ***c,*** *pH against time of a solution of 10 mM Fmoc-AVE as precursor in response to 50 mM EDC.* ***d,*** *Natural logarithm of concentration profiles of 40 mM EDC in MES buffer. A linear fit (black line) gave a k_0-value $1.3x10^{-5}$ sec^{-1}. Error bars represent the standard deviation (n=3).* ***e,*** *Concentration profiles of chemical reaction networks with 10 mM Fmoc-E as precursor and 10 mM of fuel (EDC, CMC or DIC). All fuels yielded the Fmoc-E anhydride transiently. EDC gave the highest relative yield and our studies therefore focus on this fuel.*

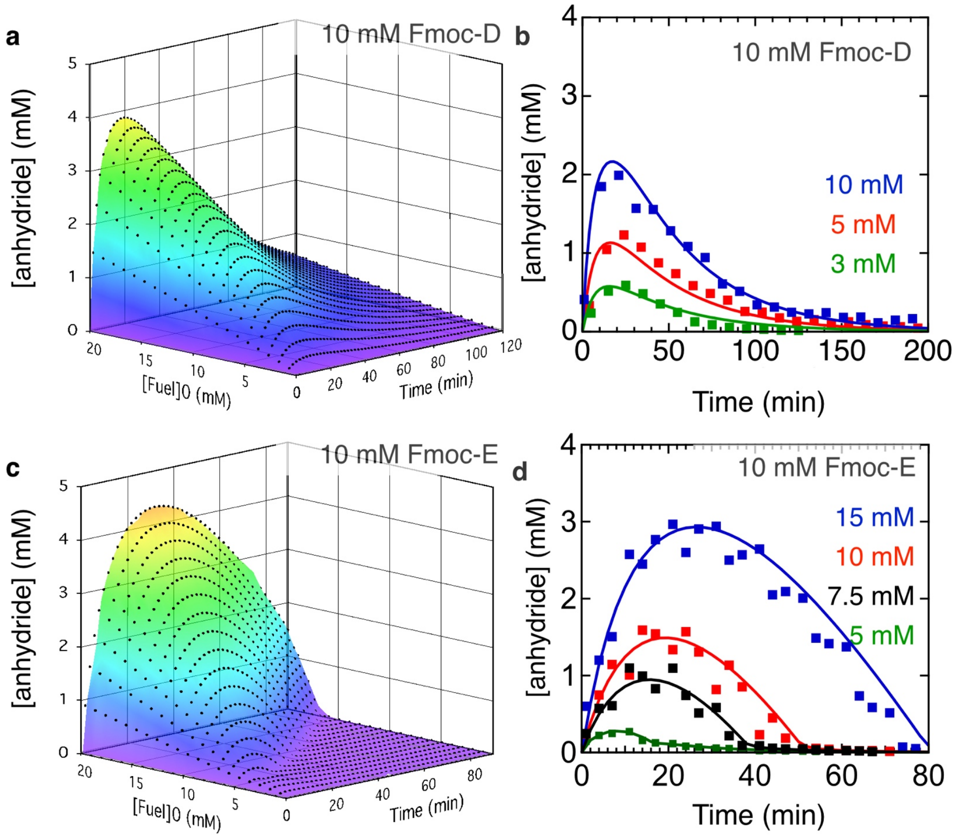

***Supplementary Figure 2: Kinetic traces of Fmoc-D and Fmoc-E with EDC. a**, Concentration profiles of chemical reaction network with 10 mM Fmoc-D as precursor and varying concentrations of fuel. The black markers represent the calculated concentration using the kinetic model, the planes represent interpolations between the model data. **b**, 2D plot of the data shown in **a** for 3, 5 and 10 mM of fuel with corresponding HPLC data. **c**, Concentration profiles of chemical reaction network with 10 mM Fmoc-E as precursor and varying concentrations of fuel in 3D plot. Colored plane is the data from the kinetic model. **d**, 2D plot of the data shown in **c** for 5, 7.5, 10 and 15 mM of fuel with corresponding HPLC data. All experiments were performed at 25°C with 10 mM precursor in 200 mM MES at pH6.*

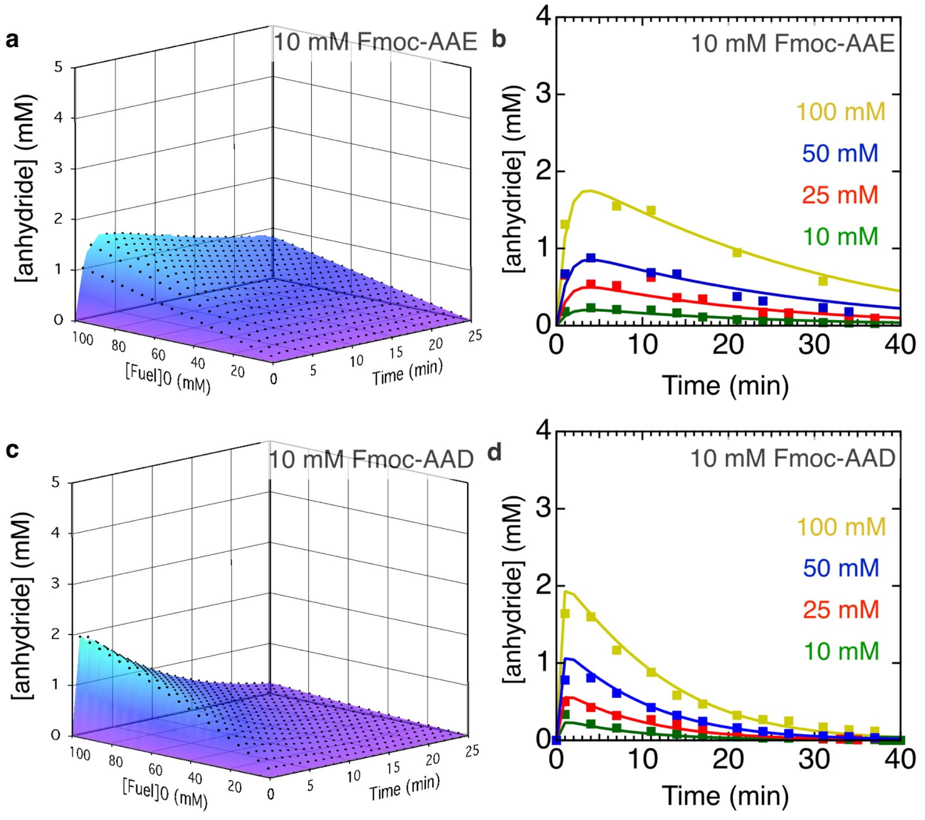

Supplementary Figure 3: Kinetic traces of Fmoc-AAE and Fmoc-AAD with EDC. a*, Concentration profiles of chemical reaction network with 10 mM Fmoc-AAE as precursor and varying concentrations of fuel. The black markers represent the calculated concentration using the kinetic model, the planes represent interpolations between the model data.* ***b****, 2D plot of the data shown in* ***a*** *for 10, 25, 50 and 100 mM of fuel with corresponding HPLC data.* ***c****, Concentration profiles of chemical reaction network with 10 mM Fmoc-AAD as precursor and varying concentrations of fuel in 3D plot. Colored plane is the data from the kinetic model.* ***d****, 2D plot of the data shown in* ***c*** *for 10, 25, 50 and 100 mM of fuel with corresponding HPLC data. All experiments were performed at 25ºC with 10 mM precursor in 200 mM MES at pH6.*

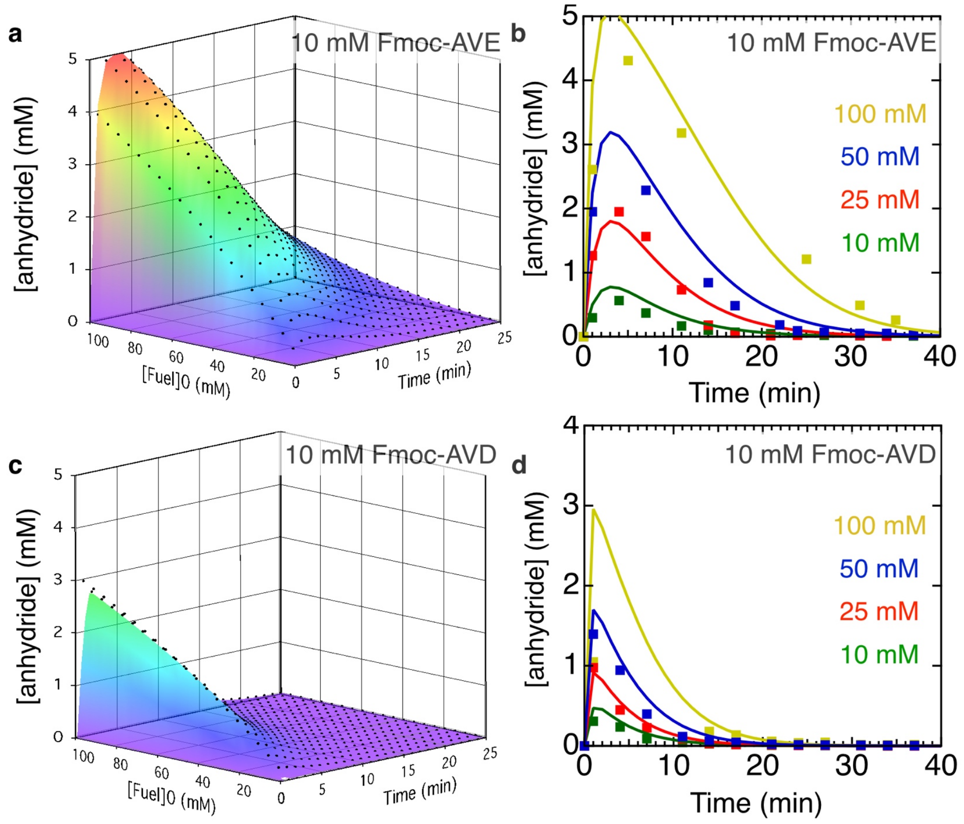

***Supplementary Figure 4: Kinetic traces of Fmoc-AVE and Fmoc-AVD with EDC. a**, Concentration profiles of chemical reaction network with 10 mM Fmoc-AVE as precursor and varying concentrations of fuel. The black markers represent the calculated concentration using the kinetic model, the planes represent interpolations between the model data. **b**, 2D plot of the data shown in **a** for 10, 25, 50 and 100 mM of fuel with corresponding HPLC data. **c**, Concentration profiles of chemical reaction network with 10 mM Fmoc-AVD as precursor and varying concentrations of fuel in 3D plot. Colored plane is the data from the kinetic model. **d**, 2D plot of the data shown in **c** for 10, 25, 50 and 100 mM of fuel with corresponding HPLC data. Note the missing HPLC data for 100 mM of fuel between 3 and 12 minutes. Data acquisition was not possible for these time points as a result of the stiff gels formed. All experiments were performed at 25°C with 10 mM precursor in 200 mM MES at pH6.*

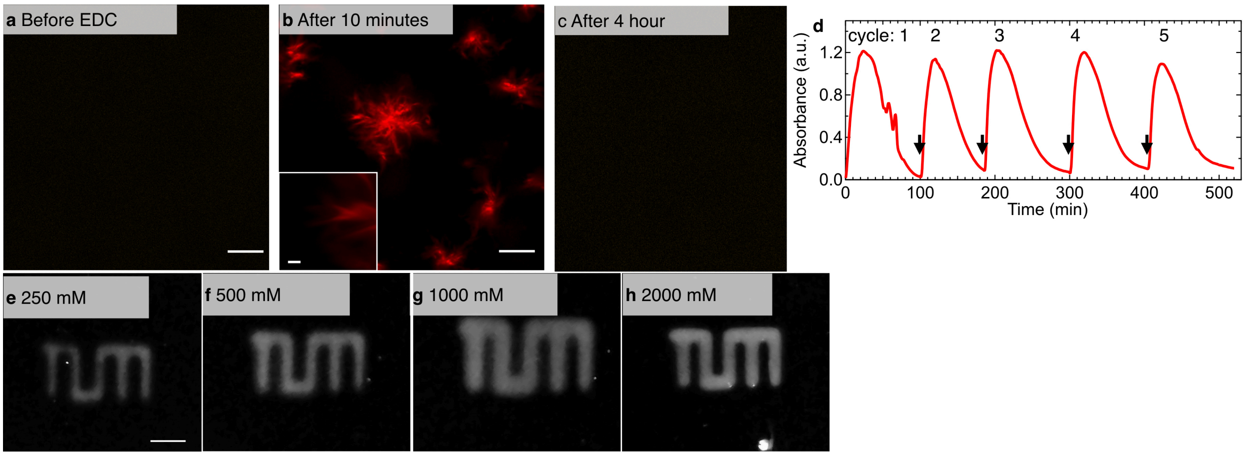

Supplementary Figure 5: Characterization of Fmoc-D in response to EDC. *Micrographs of solutions of 10 mM Fmoc-D combined with 10 mM EDC and 2.5 μM Nile Red,* ***a****, before addition of EDC,* ***b****, after 10 minutes, and* ***c****, after 4 hours (scale bars represent 10 μm, 1 μm for the inset).* ***d****, Absorbance at 600 nm as an indication of turbidity against time. 5 mM of fuel was added at each step indicated by arrows.* ***e-h****, Photographs of sprayed solutions with varying concentrations of fuel (scale bar represents 1 cm).*

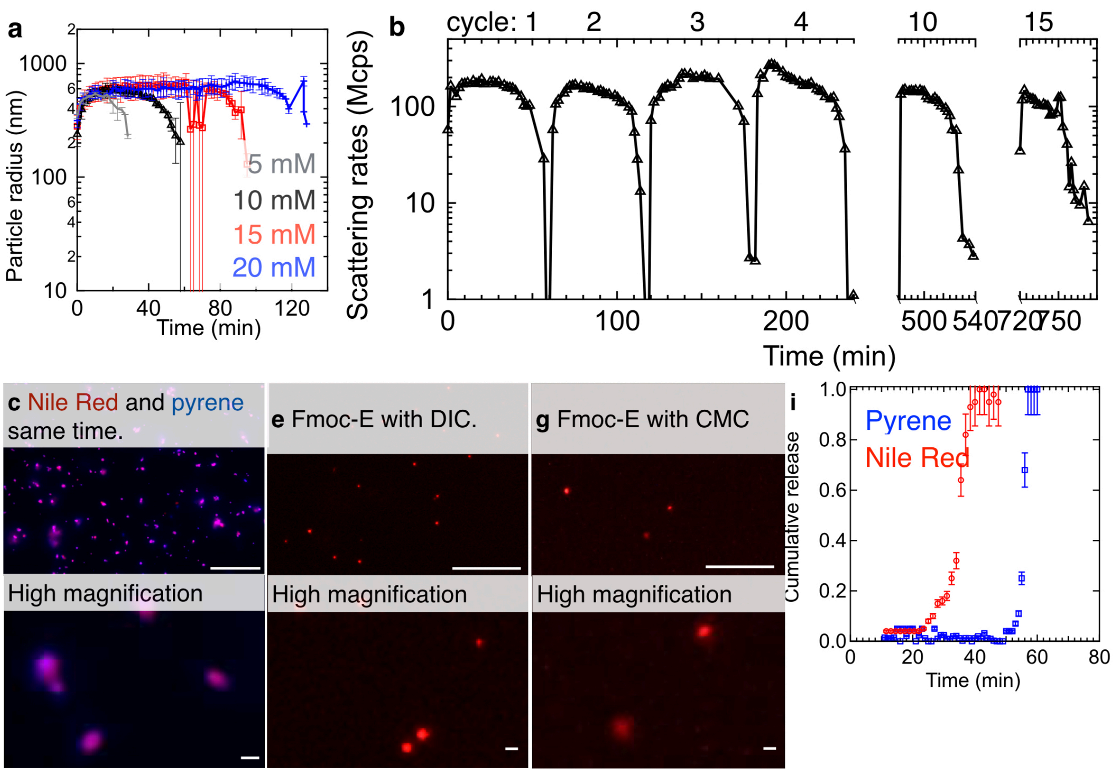

Supplementary Figure 6: Characterization of Fmoc-E in response to fuels. ***a****, Hydrodynamic radii as determined by DLS as a function of time for various initial fuel concentration. The error bars represent the standard deviation (n=2).* ***b****, DLS scattering intensity against time for four repetitive cycles of 10 mM EDC against time. New fuel was added every 60 minutes.* ***c****, Micrographs of solutions of 10 mM Fmoc-E with 10 mM EDC and 2.5μM Nile Red and 2.5 μM pyrene as dyes. Both pyrene and Nile Red were present from the beginning of the cycle. The scale bars represent 25 μm and 1 μm for the lower and higher magnification respectively.* ***d****,* ***e****, Micrographs of solutions of 10 mM Fmoc-E with 50 mM DIC (****d****) or with 50 mM CMC (****e****) and 25μM Nile Red. The scale bars represent 25 μm and 1 μm for the lower and higher magnification respectively.* ***f****, Release profiles of pyrene (blue markers) and Nile Red (red markers) of the particle described in Figure 4e. Data collection was started 10 minutes after addition of EDC. All error bars represent the standard deviation (n=2).*

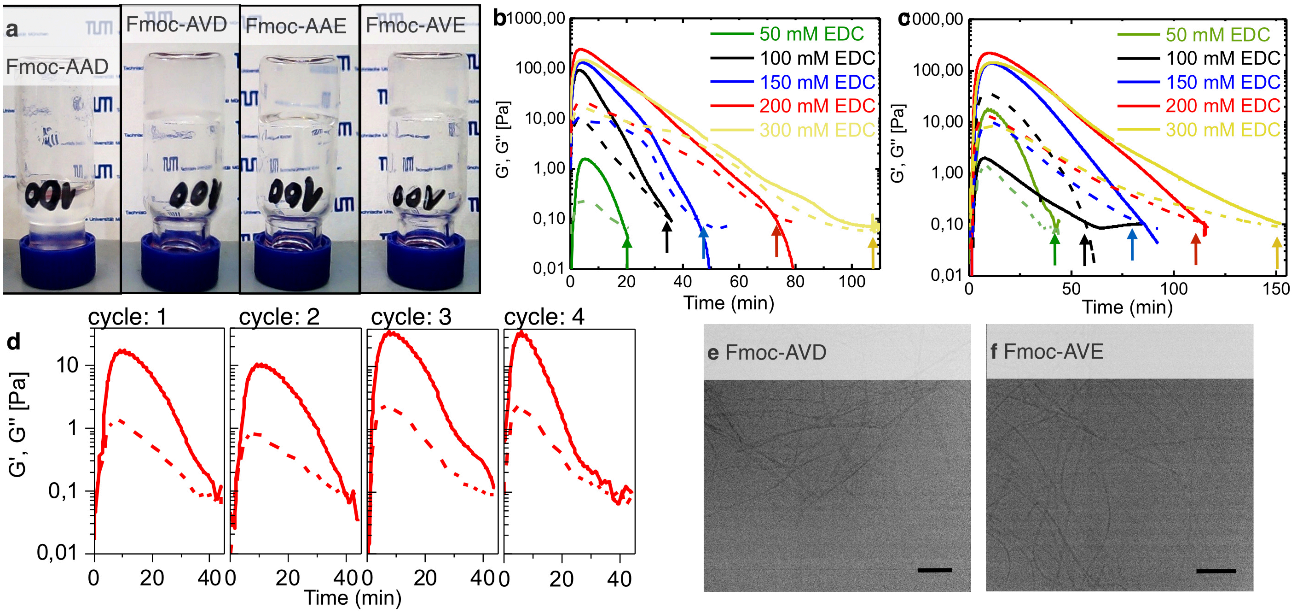

***Supplementary Figure 7: Characterization of self-assembly of Fmoc-tripeptides in response to EDC. a**, Photographs of gels of tripeptides 10 minutes after addition of 100 mM of fuel. **b**, **c**, Representative rheology time sweeps of gels formed by 10 mM Fmoc-AAD and Fmoc-AAE respectively using different EDC concentrations ranging from 50 to 300 mm. Solid lines represent the storage modulus (G'), dashed line represents the loss modulus (G''), arrows indicated the crossover point. **d**, Rheology time sweeps of gels formed by 10 mM of Fmoc-AAE and 4 cycles of 50 mM EDC addition. Gels were prepared in a separate container and placed between the two plates immediately after a new fuel addition. Fuel addition was performed every 12 hours. **e**, **f**, Cryo-TEM images for Fmoc-AVD and Fmoc-AVE 24 hour after addition of fuel (scale bars represent 100 nm).*

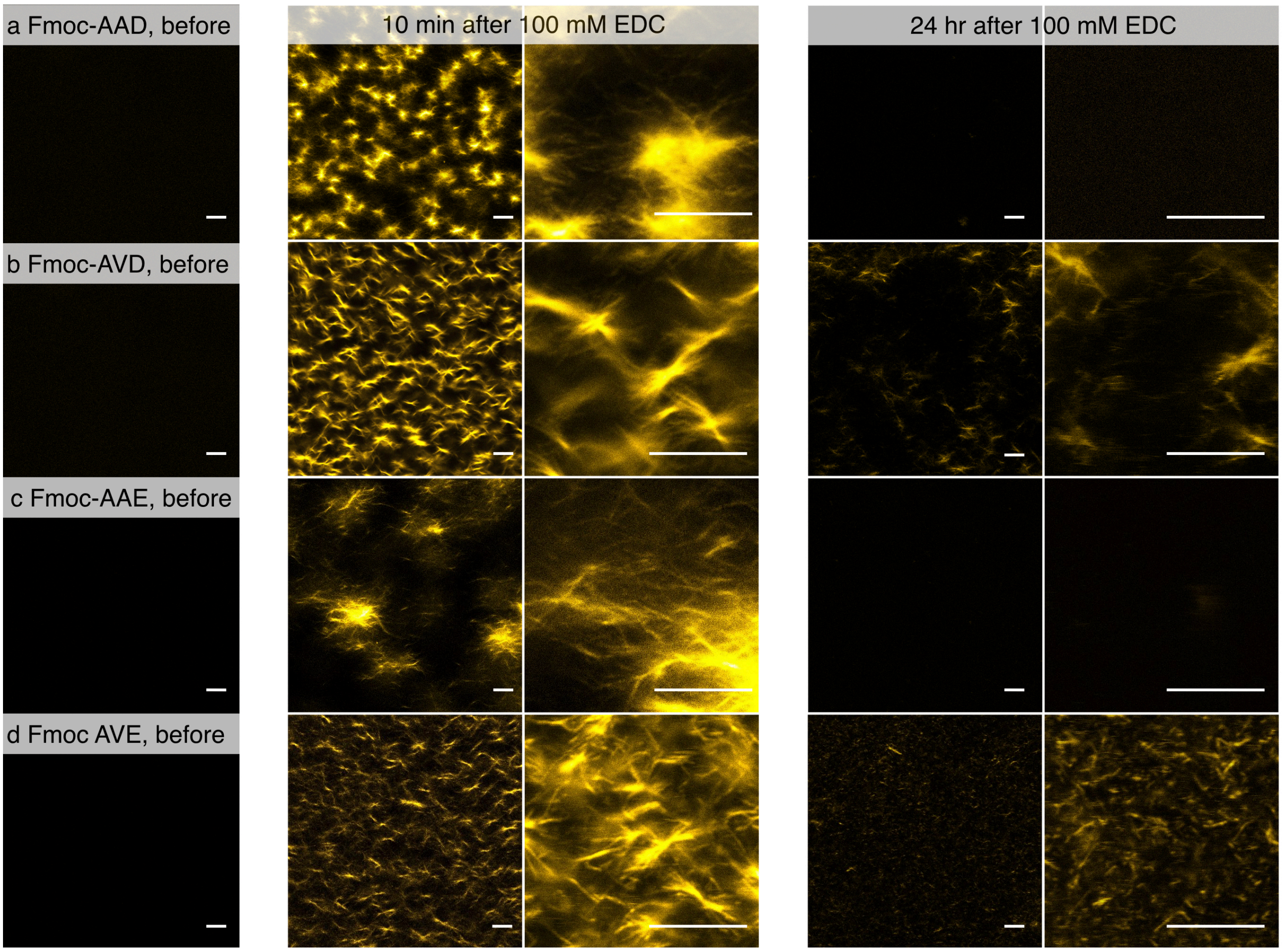

Supplementary Figure 8: Confocal micrographs of Fmoc-AA and Fmoc-AV series in response to EDC. a*, Fmoc-AAD,* ***b****, Fmoc-AVD,* ***c****, Fmoc-AAE and* ***d****, Fmoc-AVE before, 10 minutes and 24 hours after addition of 100 mM EDC. All scale bars are 10 µm.*

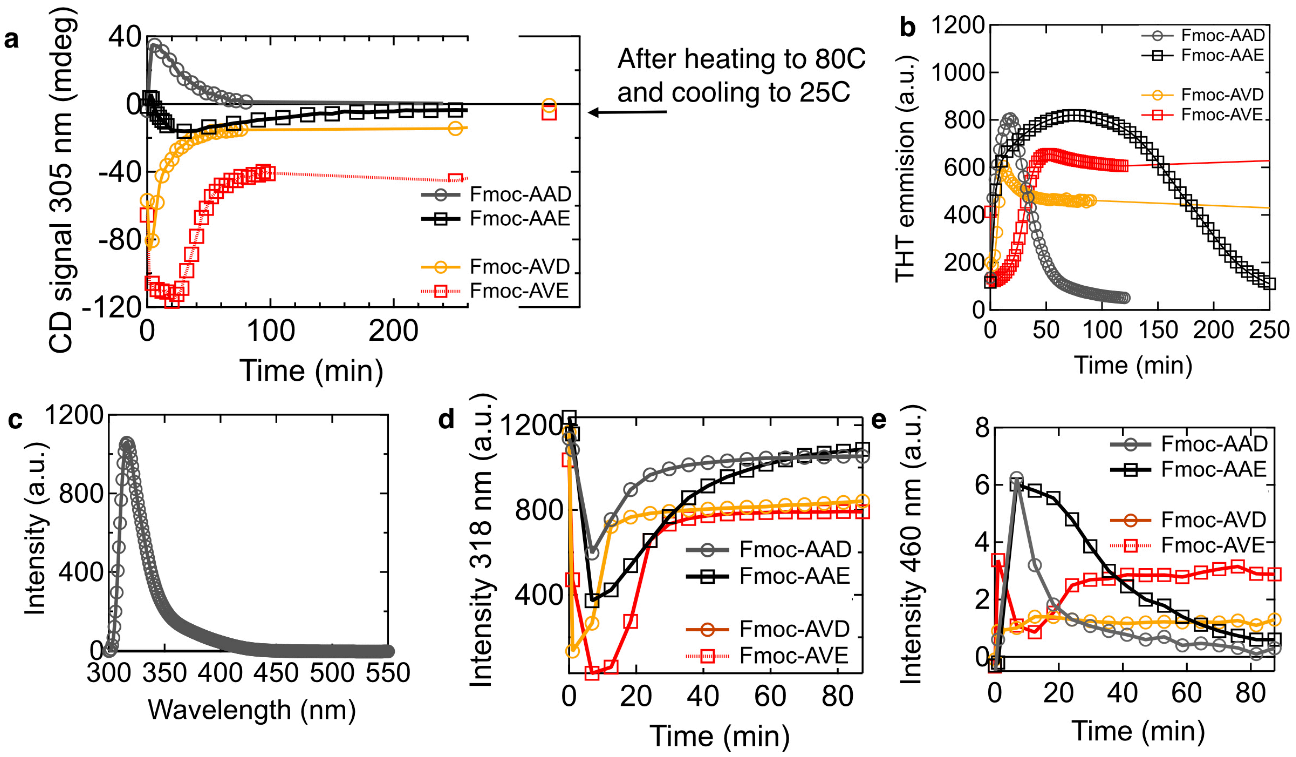

***Supplementary Figure 9: Spectroscopic characterization of self-assembly of Fmoc-tripeptides in response to EDC. a**, CD spectroscopy signal at 305 nm as an indication of self-assembly (pathlength is 1 mm). Note that the Fmoc-AV series remains in an assembled state, due to kinetic trapping within the assemblies. Only after heating and cooling is the original level obtained. **b**, THT intensity at 485 nm against time as a measure for the presence of β-sheets in the self-assembled fibers. **c**, Fluorescence emission spectrum of the Fmoc-AAD at 10 mM (excitation wavelength 285 nm). **d**, **e**, Fluorescence emission intensity at 318 nm and 460 nm respectively against time (excitation wavelength 285 nm). Note that the signal at 318 nm (corresponding to the monomeric fluorenyl group) initially decreases while the signal at 460 nm (corresponding to fluorenyl excimer) increases upon the self-assembly. Both trends demonstrate the aromatic interaction in the assembly process. Again, the Fmoc-AAX recovers to values at similar to those at the beginning of the cycle, whil the Fmoc-AVX tripeptides remain trapped.*

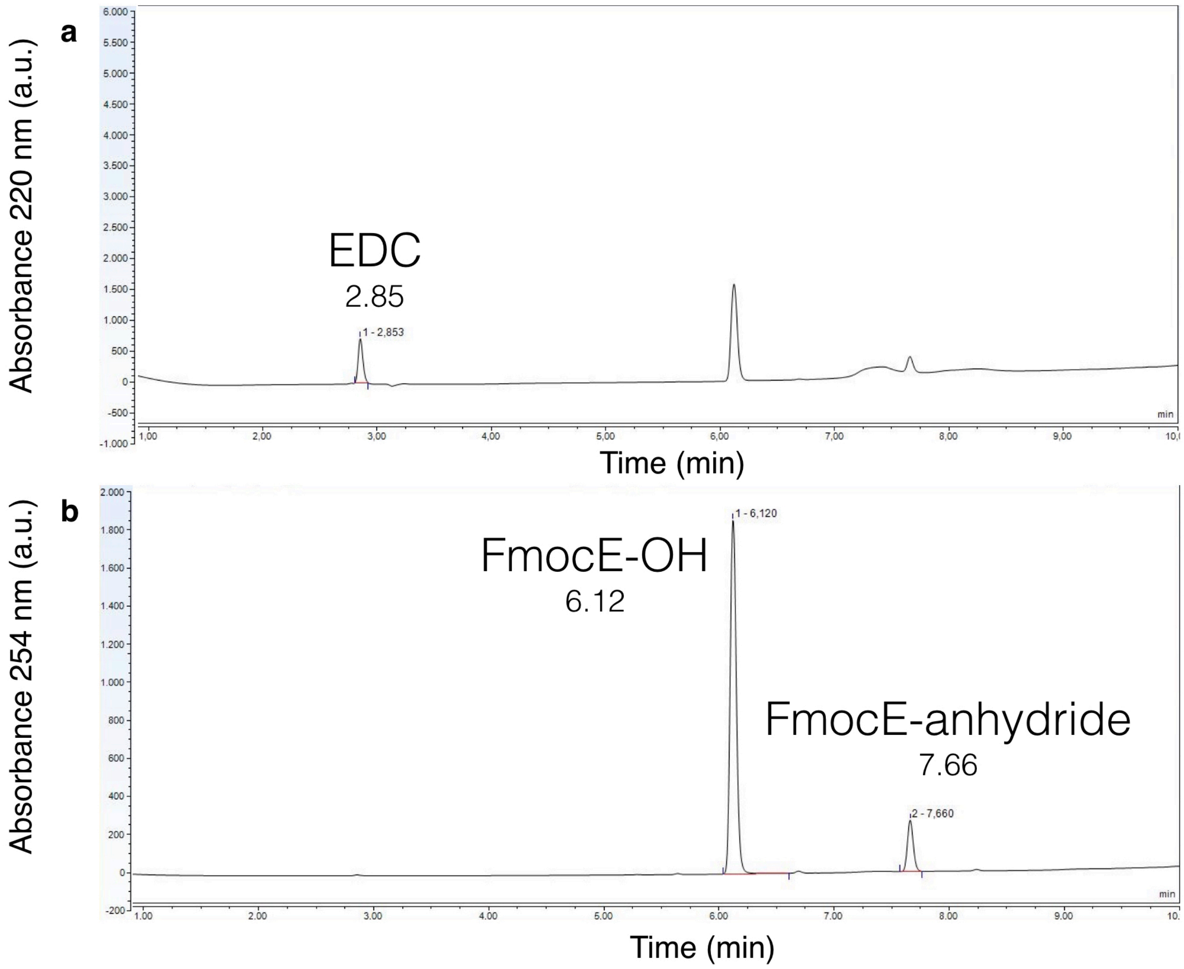

Supplementary Figure 10: Representative HPLC traces of Fmoc-E after EDC addition. *HPLC traces of 10 mM Fmoc-E after 11 minutes of EDC addition (10 mM). EDC consumption was monitored at 220 nm* ***(a)*** *while changes in precursor and anhydride concentration were monitored at 254 nm* ***(b)****. Chromatogram represents a linear gradient water: ACN from 40:60 to 2:98.*

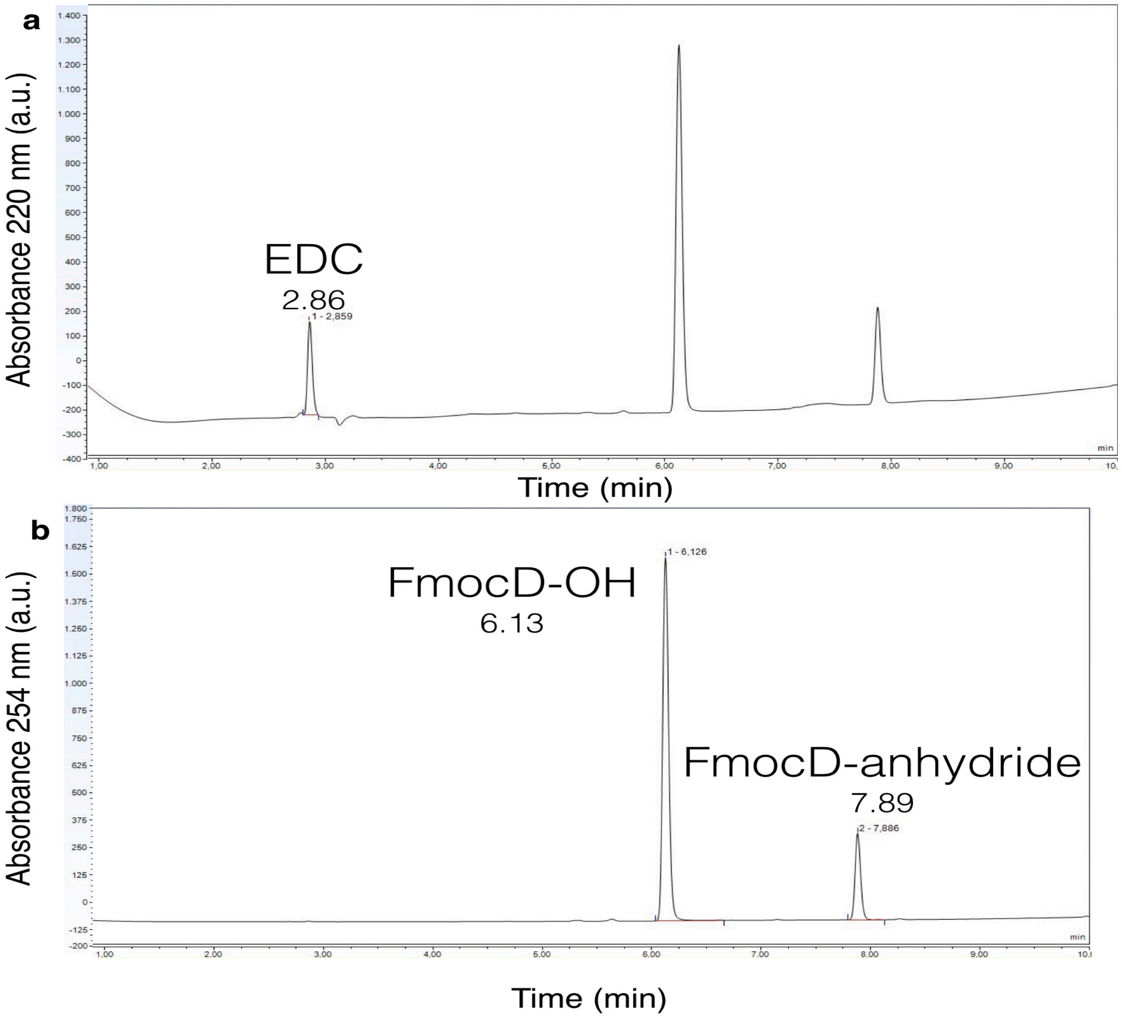

Supplementary Figure 11: Representative HPLC traces of Fmoc-D after EDC addition. *HPLC traces of 10 mM Fmoc-D after 11 minutes of EDC addition (10 mM). EDC consumption was monitored at 220 nm* ***(a)*** *while changes in precursor and anhydride concentration were monitored at 254 nm* ***(b)****. Chromatogram represents a linear gradient water: ACN from 40:60 to 2:98.*

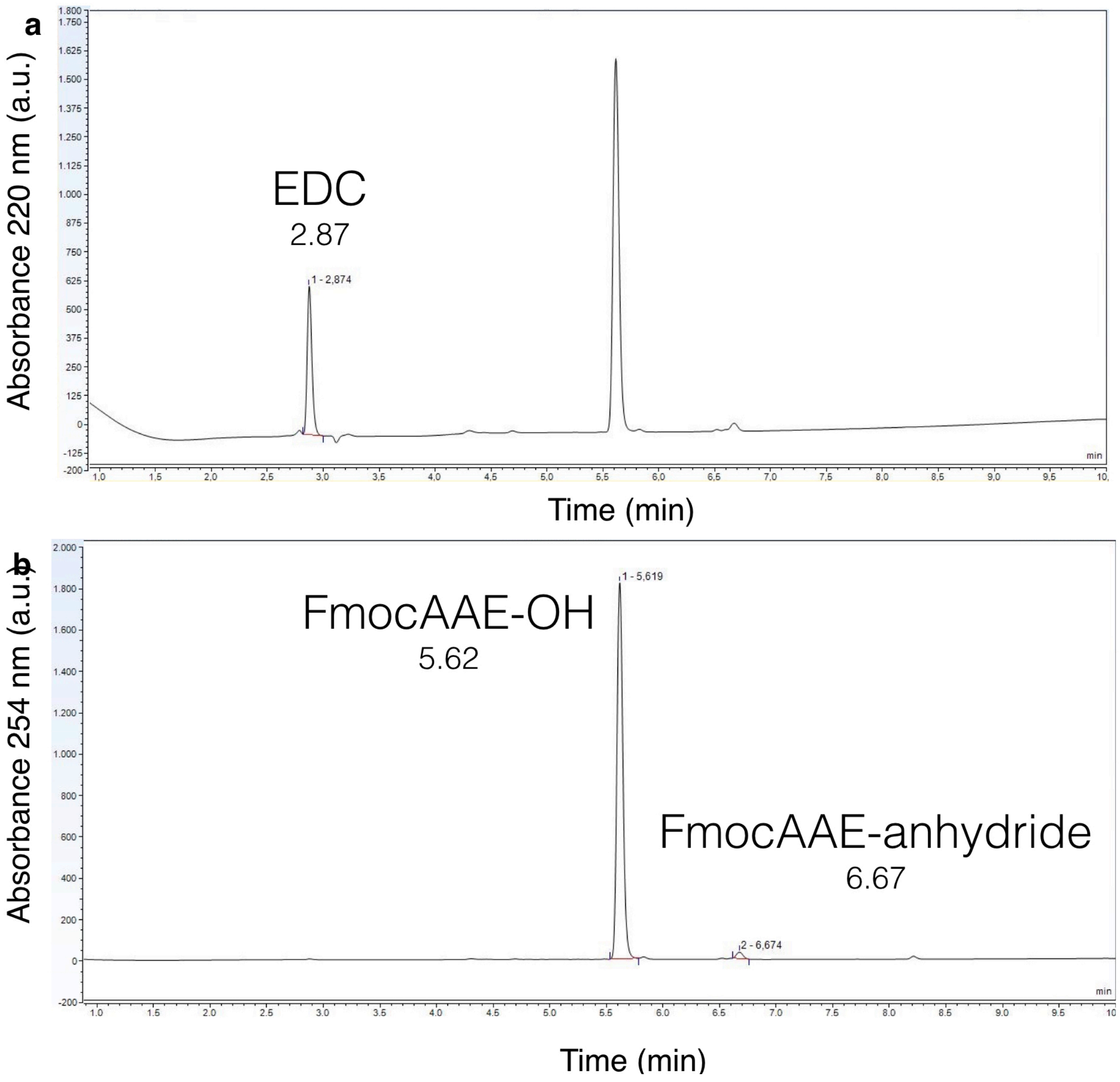

Supplementary Figure 12: Representative HPLC traces of Fmoc-AAE after EDC addition. *HPLC traces of 10 mM Fmoc-AAE after 11 minutes of EDC addition (10 mM). EDC consumption was monitored at 220 nm* ***(a)*** *while changes in precursor and anhydride concentration were monitored at 254 nm* ***(b)****. Chromatogram represents a linear gradient water: ACN from 40:60 to 2:98.*

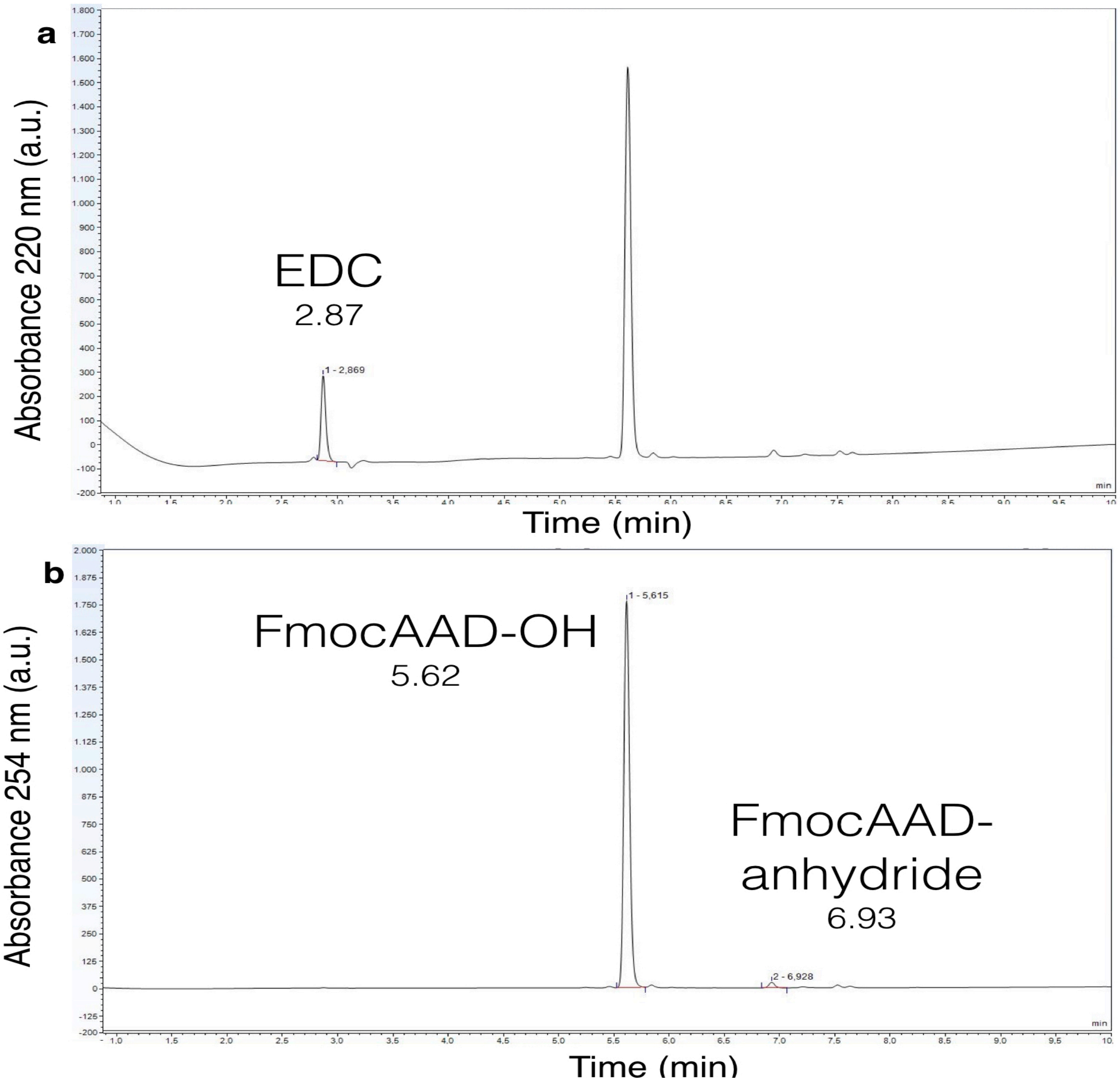

Supplementary Figure 13: Representative HPLC traces of Fmoc-AAD after EDC addition. *HPLC traces of 10 mM Fmoc-AAD after 11 minutes of EDC addition (10 mM). EDC consumption was monitored at 220 nm* ***(a)*** *while changes in precursor and anhydride concentration were monitored at 254 nm* ***(b)****. Chromatogram represents a linear gradient water: ACN from 40:60 to 2:98.*

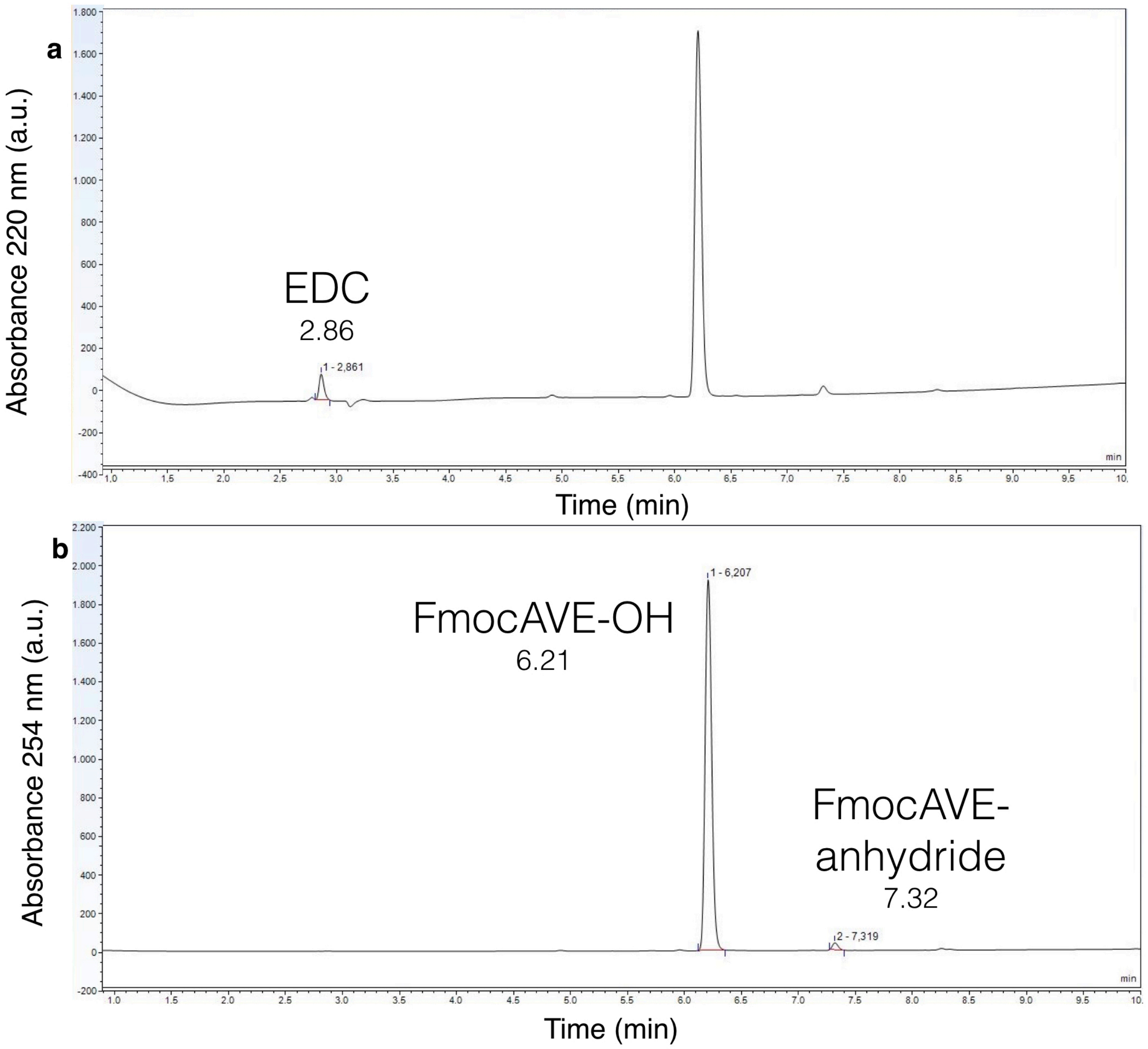

Supplementary Figure 14: Representative HPLC traces of Fmoc-AVE after EDC addition. *HPLC traces of 10 mM Fmoc-AVE after 11 minutes of EDC addition (10 mM). EDC consumption was monitored at 220 nm* ***(a)*** *while changes in precursor and anhydride concentration were monitored at 254 nm* ***(b)****. Chromatogram represents a linear gradient water: ACN from 40:60 to 2:98.*

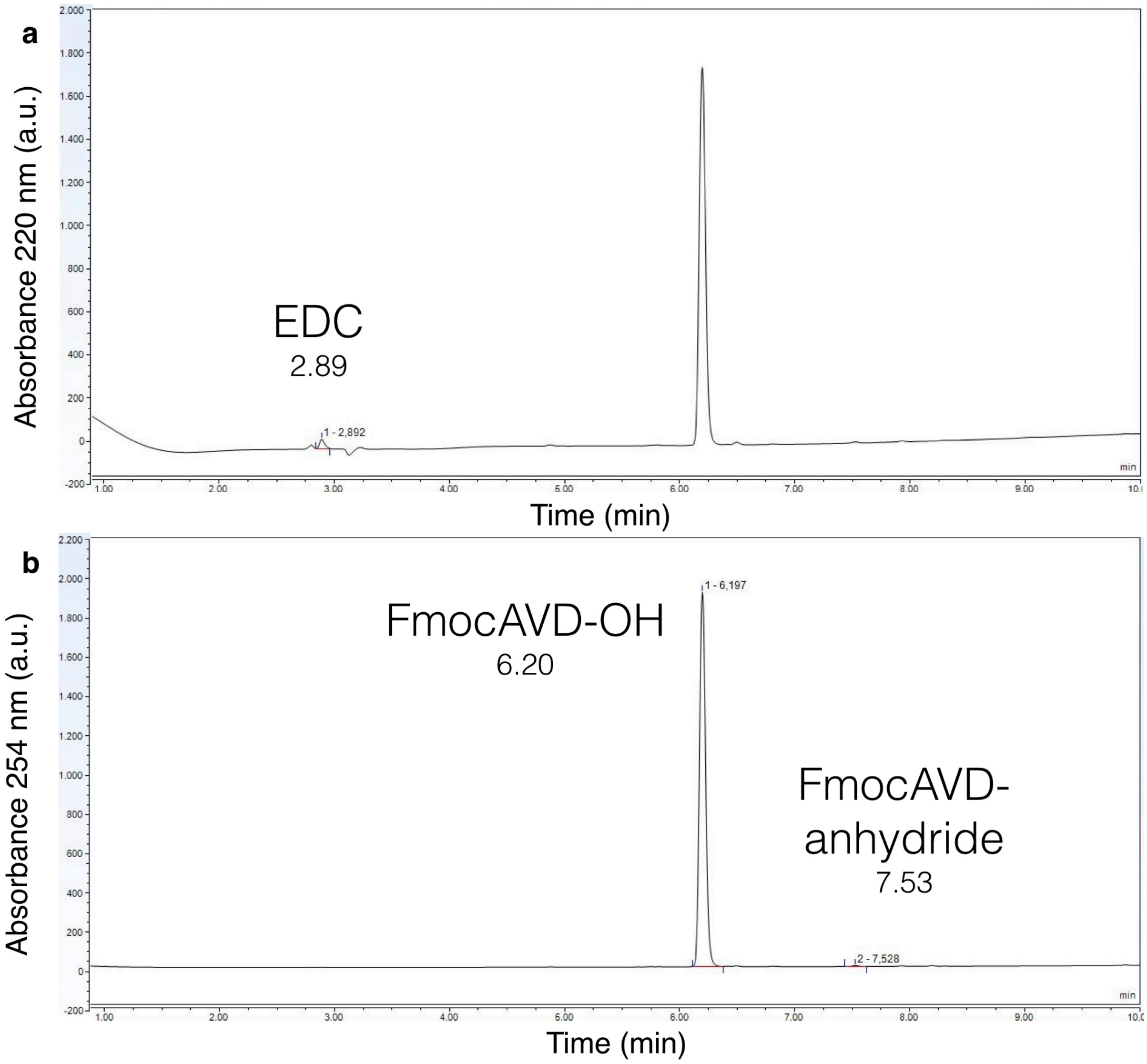

Supplementary Figure 15: Representative HPLC traces of Fmoc-AVD after EDC addition. *HPLC traces of 10 mM Fmoc-AVD after 11 minutes of EDC addition (10 mM). EDC consumption was monitored at 220 nm* ***(a)*** *while changes in precursor and anhydride concentration were monitored at 254 nm* ***(b)****. Chromatogram represents a linear gradient water: ACN from 40:60 to 2:98.*

Supplementary Methods

Peptide synthesis and purification. All peptides were synthesized using standard fluoren-9-ylmethoxycarbonyl (Fmoc) solid-phase peptide synthesis on Wang resin (100-200 mesh, 1.1 mmol/g loading). Synthesis was performed on a CEM Liberty microwave-assisted peptide synthesizer. The first amino acid coupling to the resin was accomplished by using symmetrical anhydride methodology. Briefly, a 0.2 M solution of the Fmoc-amino acid symmetrical anhydride was prepared by allowing the corresponding Fmoc-protected amino acid (FmocE(OtBu)OH or FmocD(OtBu)OH, 12 mmol) and N,N′-diisopropylcarbodiimide (DIC, 6 mmol) to react in 30 mL N,N-dimethylformamide (DMF) for 40 min. The solution was placed in the freezer for 15 min and the solid urea formed was filtered out before next step. Loading of the resin was performed using the automated peptide synthesizer. The symmetrical anhydride solution (0.2 M, 12 mL) and 4-(dimethylamino)pyridine (DMAP) solution in DMF (20 mM, 2.5 mL) were added to the pre-swollen Wang resin (0.5 mmol, 1.1 mmol/g) and heated in the microwave (30 min, 75 ºC). The coupling was repeated twice. The resin was then washed with DMF (2x10 mL). Following couplings were achieved using 4 equivalents (eq.) of Fmoc-protected amino acid in DMF, 4 eq. of DIC and 4 eq. of ethyl (hydroxyimino)cyanoacetate (Oxyma). The resin solution was then heated in the microwave (1x2 min, 90ºC). Fmoc removal was accomplished using a solution of 20% piperidine in DMF (1x2 min, 90ºC). The resin was washed with DMF (3x7 mL) between different steps. Tripeptides were cleaved from the resin using a mixture of 95% trifluoroacetic acid (TFA), 2.5% water, and 2.5% triisopropylsilane (TIPS). The solvent was removed by co-distillation with ether by rotary evaporation and dried under reduced pressure. The product was purified using reversed-phase high-performance liquid chromatography (HPLC, Thermofisher Dionex Ultimate 3000, Hypersil Gold 250x4.8 mm) in a linear gradient of acetonitrile (ACN, 40% to 98%) and water with 0.1% TFA. Purified product was lyophilized and stored at -20 °C until further use. The purity of the tripeptides was analysed by electrospray ionization mass spectrometry in positive mode (ESI-MS) as well as analytical HPLC (Thermofisher Dionex Ultimate 3000, eluted with a gradient of 0.1% TFA in water: ACN from 40:60 to 2:98 in 10 min, see below for results).

Sample preparation. Stock solutions of the precursor were prepared by dissolving the precursor in 200 mM MES buffer, after which the pH was adjusted to pH 6.0. Stock solutions of EDC were prepared by dissolving the EDC powder in MQ water. Typically, stock solutions of 1.0 M EDC were used freshly. Reaction networks were started by addition of the high concentration EDC to the peptide solution.

HPLC. The kinetics of the chemical reaction networks were monitored over time by means of analytical HPLC (HPLC, Thermofisher Dionex Ultimate 3000, Hypersil Gold 250 x 4.8 mm). A 750 µL sample was prepared as described above and placed into a screw cap HPLC vial. Every 10 minutes, samples of these solutions were directly injected without further dilution, and all compounds involved were separated using a linear gradient water: ACN from 40:60 to 2:98. In order to avoid aggregation problems during the injection, samples of Fmoc-D were continuously stirred. In the case of the gelators, gels were just slightly broken manually by shaking the vial before the injection took place.
Calibration curves for fuels (λ= 220 nm) and precursors (λ= 254 nm) were performed in triplicate in order to quantify the compounds over time. Calibration was not possible for the anhydrides due to their intrinsic instability. Instead, the absorption coefficient of their corresponding precursor was used. Measurements were performed at 25 ºC.

UV/Vis Spectroscopy. The UV/Vis measurements were carried out using a Genesys 10S (ThermoFisher) UV-VIS spectrophotometer. Samples were prepared as described above and placed in a 10 mm quartz cell (HellmaAnalytics) and stirred while acquiring data. Data was measured at 600 nm with an interval of 7 seconds.

DLS. DLS measurements on Fmoc-E solutions were performed using a DynaPro NanoStar from Wyatt with a laser wavelength of 658 nm. The MES buffered samples were measured using the disposable cuvette for DLS from Wyatt. Each measurement consisted of 5 acquisitions with an acquisition time of 10 s. For measurements and analysis the software Dynamics V7 was used. Measurements were performed at 25 ºC.

Rheology. Rheological measurements were carried out on a stress controlled rheometer (MCR 302, Antor Paar, Graz, Austria) using a plate-plate geometry (PP25, Anton Paar, Graz, Austria) and a plate separation of 0.3 mm. Samples were prepared as previously mentioned and placed between the two plates. Frequency and strain values were fixed to 1 Hz and 10 % respectively. A solvent trap was placed around the sample holder to avoid evaporation. Data was recorded at 25 ºC. The reusability experiments were performed by first preparing gels in an Eppendorf tube and, after at least 24 hours, the second, third or fourth gel was prepared in the rheometer using the above described procedure.

Confocal Fluorescence Microscopy. Confocal fluorescence microscopy was performed on a Leica SP5 confocal microscope using a 63x oil immersion objective. Samples were prepared as described above, but with 25 µM Nile Red as dye. 20 µL of the sample was deposited on the glass slide and covered with a 12mm diameter coverslip. Samples were excited with 543 nm laser and imaged at 580-700 nm.

Fluorescence Microscopy. For samples that used both Nile Red and Pyrene as dye, we used conventional fluorescence microscopy. Sample preparation was performed as described above and micrographs were acquired on a Leica DMi8 microscope using a 100x oil immersion objective.

Fluorescence Spectroscopy. Fluorescence spectroscopy was performed on a Jasco (Jasco FP-8300) spectrofluorimeter with an external temperature control (Jasco MCB-100).

In order to measure the cumulative release of Nile Red and pyrene, we used the solvatochromic properties of these dyes when free in water. Over time, as the particles hydrolysed, the Nile Red and pyrene signal decreased as it was expelled into the water phase. The signal was normalized as a measured for release of dye. Nile Red was excited at 550 nm and its emission was traced over time at 630 nm, while pyrene was excited at 330 nm and its emission traced at 384 nm. Measurements were performed at 25 ºC.

The Nile Red assay was performed on the same spectrofluorimeter as described above. Samples were directly prepared in the 10 mm quartz cuvette (Precision Cells Inc.) by mixing different concentrations of precursor (from 2.5 to 15 mM in MES 0.2 M) with Nile Red (5 µM). The fluorescence intensities were measured at 635 nm with and excitation at 550 nm.

The THT assay was performed on the same spectrofluorimeter as described above. Samples were directly prepared in the 10 mm quartz cuvette (Precision Cells Inc.) by mixing precursor (10 mM in MES 0.2M) with EDC (100 mM) and THT (5 µM). The fluorescence intensities were measured over time, every 2 minutes, at 485 nm with excitation at 450 nm.

The Fmoc assay was performed on the same spectrofluorimeter as described above. Samples were directly prepared in the 10 mm quartz cuvette (Precision Cells Inc.) by mixing precursor (10 mM in MES 0.2M) with EDC (100 mM). The fluorescence intensities were measured over time, every 5.8 minutes, from 300 to 600 nm with excitation at 285 nm.

Circular Dichroism Spectroscopy. Circular dichroism measurements were performed on a Jasco (Jasco J750) equipped with a Peltier temperature Control. Samples were prepare as described above and placed into a 1 mm quartz cell (Hellma Analytics) cuvette. Spectra were recorded from 325 to 280 nm with 0.2 nm step, 1 nm bandwidth. For single measurements, final spectra are the average of 10 accumulations at scan speed of 50 nm/min. For kinetic measurements spectra were recorded every 2 minutes with no accumulation at scan speed of 100 nm/min. Measurements were performed at 25 ºC.

Spray Coating. For the preparation of the self-erasing medium, Fmoc-D was immobilized in a 30% polyacrylamide hydrogel. Briefly, a solution of 30% (29:1) acrylamide: bisacrylamide was prepared. In this solution, 10 mM Fmoc-D and 200 mM MES was dissolved. To prepare a gel, 4mL of this stock solution was deposited in a petridish and 8µL tetramethylethylenediamine (TEMED) was added. The gelation was started by addition of 60µL 10w/w/% ammonium persulfate in water. The reaction was allowed to proceed for at least 2 hours, before EDC was spray coated or painted.

The fuel deposition via spray coating was performed using a spray gun (Harder & Steenbeck GmbH & Co. KG, Grafo T3) and oil-free nitrogen as carrier gas. The spray gun was fastened on a mount above a vertically adjustable sample stage to fixate the nozzle-to-sample distance at 15 cm. Applying a constant nitrogen pressure of 2 bar, the flow rate of the solution was set to about 15 µL s^{-1} by tuning the nozzle diameter accordingly. The spray time and thereby the amount of deposited material was defined by an electronic controller. About 300 µL of the respective solution were deposited on the substrate using 2 spray shots of 10 s duration with a short pause. 3D-printed masks were placed onto the substrates in order to create reproducible, detailed images. After the completed spray deposition, the substrates were immediately transferred to an imaging station for further analysis.

ESI. ESI-MS measurements were performed using a Varian 500 MS LC ion trap spectrometer. The samples were diluted in acetonitrile and injected into an acetonitrile carrier flow (20 µL/min).

Cryogenic-Transmission Electron Microscopy (cryo-TEM). Samples for TEM were prepared as described above. Shortly before imaging the samples were diluted a 10-fold to decrease the density of fibers in the micrographs. Cryo-TEM imaging was performed on a Jeol JEM-1400 plus operating at 120 kV. The images were recorded in a low-dose mode on a CCD camera. Quantifoil R2/2 on Cu-grid 400 mesh were used. The grids were freshly glow-discharged for 30 seconds prior to use. Preparation of the grids was performed in a FEI Vitrobot at 21 °C with the relative humidity set to 100% and the blotting force was set to -5. The sample (5 µL) was incubated for 30 seconds, blotted twice for 3.5 seconds and then directly plunged into liquid ethane that was pre-cooled by liquid nitrogen. The cryo-EM grids were transferred and stored in liquid nitrogen, and when needed, placed into a Gatan 625 cryo-specimen holder to insert into the microscope. The specimen temperature was maintained at -170 °C during the data collection.
